# Decontamination of Ti Oxide Surfaces by Using Ultraviolet Light: Hg-Vapor vs. LED-Based Irradiation

**DOI:** 10.3390/antibiotics9110724

**Published:** 2020-10-22

**Authors:** Nagore Arroyo-Lamas, Unai Ugalde, Iciar Arteagoitia

**Affiliations:** 1 Department of Medicine and Surgery, University of the Basque Country UPV/EHU, 48940 Leioa, Spain; narroyo005@ikasle.ehu.eus; 2 APERT Research Group, Department of Electronic Technology, University of the Basque Country UPV/EHU, 48013 Bilbao, Spain; unai.ugalde@ehu.eus; 3 Maxillofacial Group, Stomatology Department, BioCruces Health Research Institute, University of the Basque Country UPV/EHU, 48940 Leioa, Spain

**Keywords:** implant surface, biomaterials, implant decontamination

## Abstract

C-range Ultraviolet (UVC) mercury (Hg)-vapor lamps have shown the successful decontamination of hydrocarbons and antimicrobial effects from titanium surfaces. This study focused on surface chemistry modifications of titanium dental implants by using two different light sources, Hg-vapor lamps and Light Emitting Diodes (LEDs), so as to compare the effectivity of both photofunctionalization technologies. Two different devices, a small Hg-vapor lamp (λ = 254 nm) and a pair of closely placed LEDs (λ = 278 nm), were used to irradiate the implants for 12 min. X-ray Photoelectron Spectroscopy (XPS) was employed to characterize the chemical composition of the surfaces, analysing the samples before and after the lighting treatment, performing a wide and narrow scan around the energy peaks of carbon, oxygen and titanium. XPS analysis showed a reduction in the concentration of surface hydrocarbons in both UVC technologies from around 26 to 23.4 C at.% (carbon atomic concentration). Besides, simultaneously, an increase in concentration of oxygen and titanium was observed. LED-based UVC photofunctionalization has been suggested to be as effective a method as Hg-vapor lamps to remove the hydrocarbons from the surface of titanium dental implants. Therefore, due to the increase in worldwide mercury limitations, LED-based technology could be a good alternative decontamination source.

## 1. Introduction

Titanium (Ti) dental implants have been widely used as prosthesis anchors since Brånemark and other colleagues discovered osseointegration [1,2]. However, despite their high survival rate predictability of up to 98%, the total implant area covered by bone (or bone–implant contact percentage) remains far from the ideal 100% [3,4,5,6]. The success and long-term survival of dental implants are influenced by a wide range of factors, such as the mucosal thickness, prosthesis connections and the positioning of the implant, among others [7,8,9,10]. Most complications are associated with the lack of enough osseointegration due to infection, caused by bacterial biofilms’ formation. Indeed, the bacteria are capable of colonizing the implant surface, causing peri-implant mucosa inflammation and progressive loss of supporting bone, and finally, leading to its failure [11]. In this context, the presence of optimal mucosa thickness, with a good soft-tissue sealing, can reduce risk of inflammation and biofilms’ formation [12]. In order to achieve a good soft-tissue sealing and osseointegration, fibroblasts, osteoblasts, and other cells, need to adhere to the surface of the implant and abutment before bacteria, covering the surface with a cellular layer, and therefore, competing with cells to reduce bacterial attachment from the surface [13,14].

In particular, the microscopic characteristics of the surface such as topography, wettability and chemical composition have been proven to be the major matters affecting the bone–implant interactions and bacterial attachment [15,16,17,18,19,20]. Moreover, low surface free energy (SFE) implant surfaces, known as superhydrophilic surfaces, reduce the biofilm formation, since many of the early colonizer bacteria (e.g., *Actinomyces israelii* and *Streptococcus sanguinis*) are hydrophobic, and therefore, the hydrophilic surfaces attract highest numbers of hydrophilic bacteria [19,21].

Specifically, the chemical composition of the surface of Ti implants is one of the most crucial factors for successful osseointegration and anti-microbial effects. Despite being sterilized in the original packaging, the TiO_2_ layer surrounding the Ti implants gets contaminated by organic impurities, such as hydrocarbons present in the atmosphere, in the transit from its manufacture to the placement in the oral cavity bedding. This process is called biological ageing of titanium, and the presence of hydrocarbons contribute to the bacterial adhesion binding to the hydrophobic molecules on the bacterial surface [22,23,24,25]. As a result of these complications, several procedures are being implemented to create antimicrobial and osteoconductive implants, such as to store the implants in liquid (e.g., distilled water), to purposely thicken the TiO_2_ layer, to apply nonthermal atmospheric-pressure plasma (NTAPP) techniques, or to irradiate them by using ultraviolet light [24,26,27,28].

In fact, recently, it has been reported that C-range ultraviolet light (UVC) irradiation emitted by mercury vapor lamps can reverse the biological ageing of titanium, in a process known as photofunctionalization [29,30,31]. This method is based on removing the hydrocarbons by two possible mechanisms: inducing the photocatalytic activity of the TiO_2_ layer as well as by the hydrocarbon’s direct decomposition. In this regard, via titanium photocatalytic activity, reactive oxygen species (e.g., -OH, O_2_^−^, -H_2_O_2_) are generated, which are responsible for the decomposition of the outer membrane of microorganisms [32,33]. Furthermore, the decontamination of hydrocarbons from the surface is associated with an increase in implant stability quotient (ISQ), bone–implant contact (BIC), an enhancement in the attachment, proliferation and differentiation of cells and a reduction in the bacterial attachment [34,35,36,37,38,39].

Nevertheless, in 2013 the worldwide treaty of the Minamata Convention on Mercury was negotiated in the United Nations (UN) Environment Program [40]. The main objective of this agreement is to protect human health from the harmful effects caused by emissions and releases of mercury (Hg). As a result, Hg-lamp production and commerce must halt by 2020, which makes it necessary to use other types of light sources. One obvious alternative is to use Light Emitting Diodes (LEDs), a well-known and mature lighting technology, by choosing commercial devices which operate in the UVC range. Currently, no published studies have been found in the databases that compare at the same time the efficacy of both light sources in the decontamination of hydrocarbons from Ti dental implants.

We hypothesized that UVC light emitted by LED-based sources successfully eliminate contaminant hydrocarbons from the Ti oxide surfaces. The main purpose of the present in-vitro study was to compare the effectivity of two different UVC light photofunctionalization technologies, Hg-vapor and LED-based sources, in decontaminating the surface chemistry of commercially available titanium dental implants.

## 2. Results

The XPS measurements of all detected elements before UVC light treatment were shown in terms of relative atom concentrations (C at.%). Although the major elements presented on the implant surface were carbon (C), oxygen (O) and titanium (Ti), other elements such as aluminum (Al), silicon (Si), nitrogen (N), vanadium (V) and calcium (Ca) were also observed. In addition, a fluoride deposit appeared in the samples.

**Figure 1:**
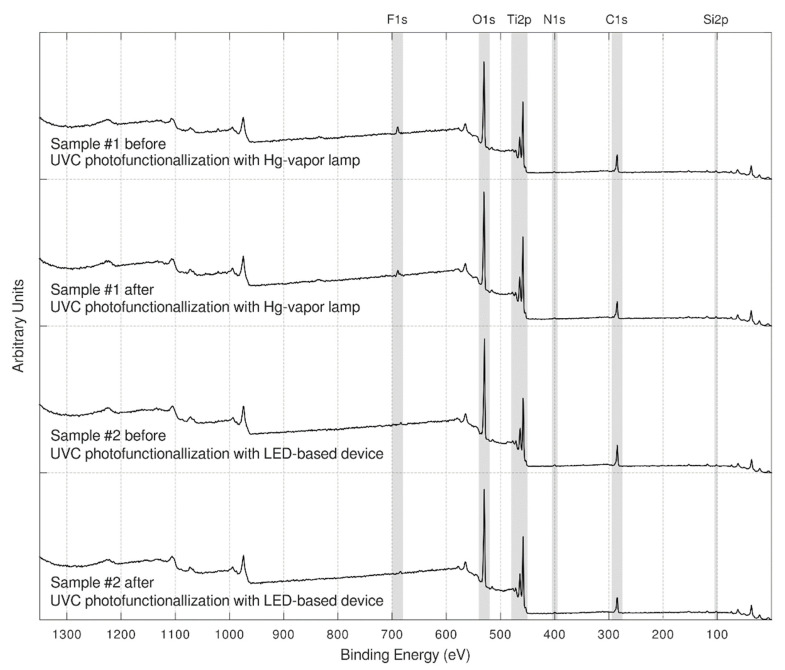
XPS full-range spectra: before and after the lighting treatment.

In Figure 1, the XPS full-range spectra (wide scan) of each sample, before and after the lighting treatment, are presented. These survey spectra showed close similarities between samples.

As far as carbon concentration is concerned, samples showed a higher carbon contamination around 26 C at.% before photofunctionalization, whereas after the UVC light treatment, a decrease in the carbon was observed in the Hg-vapor lamp device and the LED-based device, with 23.4 C at.% (Table 1). Hence, while the atomic concentration of the carbon was decreasing, the atomic concentration of O and Ti were remarkably increased.

**Figure 2:**
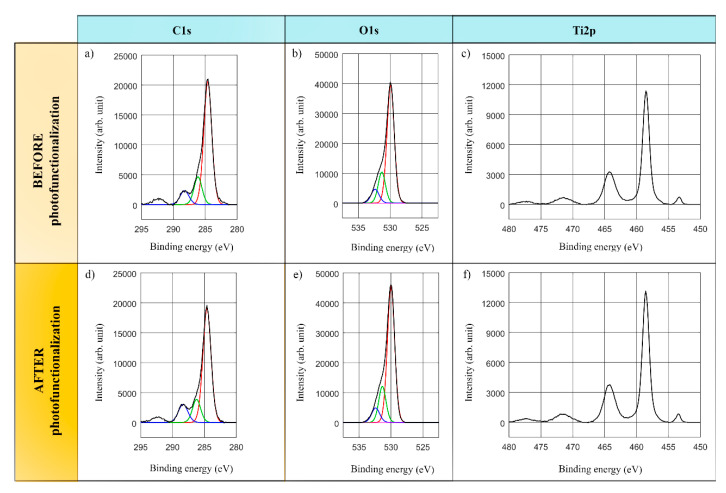
**Deconvoluted XPS with lines analysis and binding energies: Hg-vapor lamp device.** C1s, O1s and Ti2p spectra for sample #1 before (**a**–**c**) and after (**d**–**f**) photofunctionalization.

**Figure 3:**
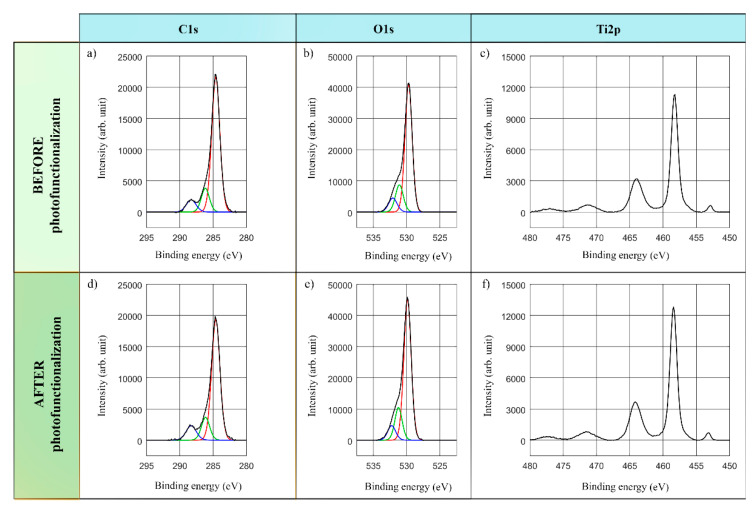
**Deconvoluted XPS with lines analysis and binding energies: LED-based device.** C1s, O1s and Ti2p spectra for sample #2 before (**a**–**c**) and after (**d**–**f**) photofunctionalization.

This study was mainly focused on C, O and Ti elements detected on the surfaces of the implants. In this regard, Figure 2 and Figure 3 present the stoichiometrical deconvolutions of C1s, O1s and Ti2p of two devices, before and after the lighting treatment.

The C1s spectra deconvolution of the treated samples consists of three components: the main peak at energy 284.6 eV concerning the hydrocarbons’ C atomic concentration in the Hg-vapor and LED-based devices, whereas the second and third components correspond to the C-O and C=O bonds in Figure 2a,d and Figure 3a,d.

The O1s spectral profile in Figure 2b,e and Figure 3b,e highlights the three main components. The component with the highest intensity at 529.9–530 eV represents TiO_2_ bonds, whereas the second and third components could be ascribed to other kind of bonds such as Ti-OH, C=O…, which could not be dependably determined because of the non-significant peaks.

Finally, the analysis of the Ti doublet peak core lines, reported in Figure 2c,f and Figure 3c,f, involves two components. The binding energy labelled Ti2p at about 458.4–458.6 eV corresponds to TiO_2_ compounds. Thus, not only was the presence of the TiO2 determined by the main peak of Ti2p in all the measurements, but the metallic form of the titanium was also confirmed at 453.8–453.9 eV. Lastly, a double satellite peak could be observed at 471.5 eV.

## 3. Discussion

In the present study, two varying devices based on different UVC sources were used to assess the successful decontamination of hydrocarbons from the surfaces of titanium dental implants, which is by far considered the most causal element related to the biological ageing and bacterial attachment. After 12-min UVC photofunctionalization with Hg-vapor and LED-based sources, both samples showed a clear reduction of about three percentage points of the atomic concentration of C, correlated with an enhancement of the atomic concentration of O and Ti. Consequently, taking into consideration the limitations of current mercury regulations, it is encouraging to see that LED-based technology produces equal results.

By and large, implants resulted in mainly C, O and Ti major elements’ presence. Additionally, other elements were detected, such as Al and V, which are related to the most common titanium alloy (Ti-6Al-4V) used for dental implants [41]. Moreover, the small silicon (Si) content would be an organosilicon compound, resulting from organic silicon residues, obtained during the cleaning procedures or lubricants used in manufacturing. There was also a tiny N peak detected at 401.5 eV, which indicated the presence of a frequent contaminant on industrial products, specifically, small concentrations of ammonium or organic nitrogen-containing species. Besides, Ca would come from the rinsing water used during their production. Finally, F presented on them have linked with the vast array of surface modification treatment methods used. In particular, the presence of F content indicated that the samples had been hydrofluoric acid etched and the rinsing procedures had not removed all the fluoride from the surface [42,43].

The XPS deconvolution spectral data of C1s showed the presence of hydrocarbon in all the implants, whose elimination from the surfaces was observed after the UVC lighting treatment. Indeed, it is known that there is a close relation between the reduction of hydrocarbon and an increase in Ti ratio, since the direct photolysis of hydrocarbon can improve the early healing bone–implant interactions by stimulating the osteoconductivity capacity and antimicrobial effects [44,45]. In addition, several works proved that chemistry composition of Ti implants plays a crucial role for successful osseointegration and antibacterial adhesion [2,43,46,47,48,49,50,51].

Concerning the characteristics of the surfaces, topography and alloys need to be taken into account. Therefore, machined surfaces, in comparison with rough surfaces, demonstrated more carbon content [33]. Additionally, regardless of the type of alloy used, both Ti and Ti6Al4V showed similar antimicrobial responses after being subjected to ultraviolet light. [50,52].

On the other hand, not only the osteoblasts conductivity was induced by ultraviolet treatment on Ti surfaces, but also the attachment, proliferation and viability of fibroblasts, which are responsible of a good soft-tissue sealing [53,54].

Although different wavelength ranges of UV irradiation are known to be effective to trigger chemical modifications, only the UVC wavelength (i.e., λ ≤ 280 nm) resulted in a remarkably better enhancement in the biological activity and greater reduction in bacterial attachment [33,36,53,55]. Longer wavelengths, such as UVA (i.e., λ ≥ 315 nm), have proven their effectivity in the decontamination of carbon and bacteria. However, the biological effects have only been shown in UVC when is compared with UVA [36]. Besides, UVA demonstrated a lower reduction in carbon content, lower hydrophilicity induction capacity and fewer antimicrobial effects than UVC [19,45,53,56]. Furthermore, most in-vitro and in-vivo studies that used Hg-vapor lamps in UVC photofunctionalization not only shown an increase in the osteoblastic activity in the early healing periods, but also a transformation in the wettability behavior from hydrophobic to superhydrophilic and antibacterial effects [45,57,58,59,60,61]. Nevertheless, contrary to all the aforementioned papers, some research works do not observe significant effects of BIC and ISQ on photofunctionalized titanium dental implants after 9 months in minipigs [62].

As far as the duration of the irradiation is concerned, it was chosen to be 12 min, like several other published works [31,63]. These works reported decontamination results similar to ours, which were ascribed to the reverse results of the biological ageing of titanium and confirm the effectivity of UVC light treatment by using different light sources. Nonetheless, longer durations, such as 15 min, 24 h or 48 h, also yielded sample decontamination, but are less practical in clinical dentistry [24,35,36,58,64,65,66].

The present study may provide a novel way of eliminating contaminant hydrocarbons from Ti oxide surfaces by using LED-based UVC sources. In this context, and bearing in mind the Minamata Convention on Mercury, the obtained results are encouraging to propose an alternative source of Hg-vapor lamps. Notwithstanding, although both devices show many similarities, the obtained results require careful interpretation, since only a single action protocol was used. Besides, future studies need to be conducted in order to determine the UVC effects with different exposure times.

Furthermore, it can be considerably difficult to compare all the studies, due to the wide range of different irradiation treatment durations, types of samples and preparations, as well as different photofunctionalization devices in the market.

## 4. Materials and Methods

### 4.1. Sample Selection

Original screw-type commercially available Ti dental implants (Sterioss THD; Anaheim, CA, USA) were investigated. The length of all implants was 16 mm and the diameter 3.8 mm, according to the description of the manufacturers.

### 4.2. Ultraviolet Irradiation Regimes

One of the Ti dental implants was placed inside a Hg-vapor lamp sterilizer-device (λ = 254 nm) (Sanitizer SG-111; Ningbo Seago Electric Co, Zhejiang, China), whereas the other was placed inside a custom designed LED-based device (λ = 278 nm) (LEDs: LEUVA66B00HF00; LG Innotek, Seoul, Korea). All the titanium specimens were treated by UVC light for 12 min, calculated by a digital timer and under ambient conditions. The distance from the light source was estimated to be 2 cm in both devices. The light power source used in both devices was 2 mW.

### 4.3. X-ray Photoelectron Spectroscopy (XPS)

After removing their original packages, both samples were placed on the metal deck of the XPS equipment, and introduced into it, in order to analyse the surface chemistry, prior to the UV treatment. For each implant sample, three evaluation areas were analysed by XPS to ensure homogeneity, between the 4th and 5th threads, 6th and 7th threads, and 19th and 20th threads of the implant.

The measurements were made using XPS equipment (SPECS System, Berlin, Germany) with a Phoibos analyzer 150 1D-DLD and monochromatic Al Kα (1486.7 eV) X-ray source. The spectral data were analysed under vacuum pressure of 5 × 10^−5^ mbar. The measured area was always 1 mm × 3 mm, and the exit angle was 90°. Then, a wide scan was first performed to determine the elements present on the surface (step energy 1 eV, dwell time 0.1 s, pass energy 80 eV); and a detailed narrow scan was performed next, focusing on the major elements detected (step energy 0.1 eV, dwell time 0.1 s, pass energy 30 eV).

**Figure 4:**
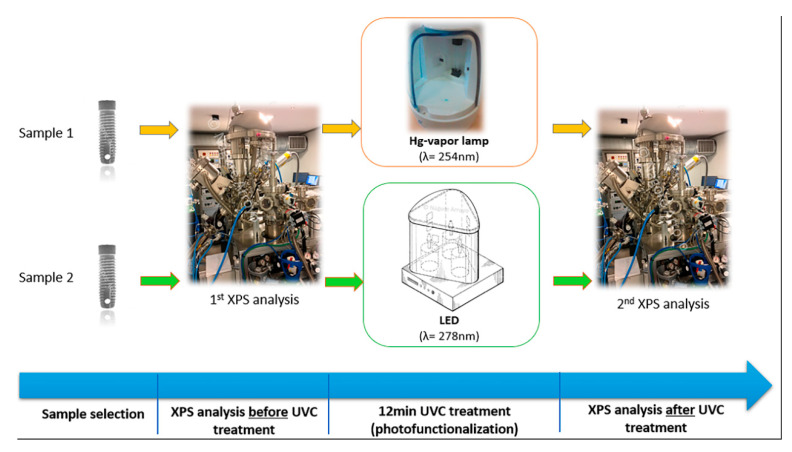
Process of photofunctionalization according to our protocol.

The samples were then UVC-treated (i.e., photofunctionalized) as described in Section 4.2, and the XPS analyses were repeated exactly in the same way as described above, so the same information was available for the pre-treated and post-treated samples. Figure 4 summarizes the whole process carried out.

### 4.4. Statistical Analyses

The XPS spectra were adjusted by using the CasaXPS 2.3.16 software (Casa Software Ltd.; Teingmouth, Devon, UK), which models the Gauss–Lorentzian contributions, after a background subtraction (Shirley). Besides, a descriptive analysis and deconvolutions of the detected elements was made.

## 5. Conclusions

The obtained results in this study suggest that LED-based UVC photofunctionalization (λ = 278 nm) is as effective a method as Hg-vapor lamps (λ = 254 nm) to remove the hydrocarbons from the surface of titanium dental implants. Moreover, the results of the present work make it possible to show the chemical modifications after UVC treatment associated with the reverse of the biological ageing of titanium and antimicrobial effects. As a result, this UVC-induced chemical change would involve positive biological and antibacterial effects reported in the scientific literature.

## Figures and Tables

**Table 1:** XPS analysis of implant treated by Hg-vapor lamp device and LED-based device (before and after UVC treatment): Atom concentration rate (% at). These are the results of the central point between the 6th and 7th thread, since the data of the other measurements do not differ.

Elements	Binding Energy	Hg-Vapor Lamp Device		LED-Based Device	
		Before (% at rel)	After (% at rel)	Before (% at rel)	After (% at rel)
C	284.6–292.4	26.5	23.4	26.6	23.4
O	529.9–532.4	48.0	50.1	49.8	51.4
Ti	458.5–460.0	16.3	17.5	17.3	18.5
F	684.6	3.7	3.4	0.6	0.7
N *	401.5	1.0	0.9	1.1	1.1
Al	73.8	2.1	2.3	2.4	2.7
Si	102.0	1.7	1.7	1.4	1.5
V *	515.1	0.5	0.7	0.6	0.6
Ca *	347.7	0.1	-	0.2	-

* Spectra close to noise.
